# Bullous Pemphigoid Complicated by Sepsis

**DOI:** 10.7759/cureus.28765

**Published:** 2022-09-04

**Authors:** Dipal Shah, Aanchal Shah, Viet S Nguyen, Michael Falgiani, Latha Ganti

**Affiliations:** 1 Emergency Medicine, HCA (Hospital Corporation of America) Florida Ocala Hospital, Ocala, USA; 2 Emergency Medicine, University of Central Florida College of Medicine, Ocala, USA; 3 Medicine, Florida State University, Talahassee, USA; 4 Emergency Medicine, Envision Physician Services, Plantation, USA; 5 Emergency Medicine, University of Central Florida College of Medicine, Orlando, USA

**Keywords:** sub-epithelial, pruritis, blistering, blisters, bulbous pemphigoid

## Abstract

Patients with bullous pemphigoid face many challenges when managing their disorder, one of which is balancing medication with their ailments. Because the patient population with bullous pemphigoid are primarily elderly, the current first-line treatment of corticosteroids tends to increase their rates of morbidity and mortality. During the acute process of the disease, providers must also consider the increased chance of infections caused by the opening in the skin. These patient cases are often complicated further by secondary symptoms such as pruritis and pain. Here we present a case in which we provided care to a 38-year-old female with a history of bullous pemphigoid and multiple medical problems who presented to the emergency department with nausea, vomiting, fevers, abdominal pain, and blisters on her forearm. Due to concern for sepsis and her past failure of outpatient therapy, the patient was hospitalized and treated for her possible infection, bullous pemphigoid, nausea, and pain.

## Introduction

Bullous pemphigoid (BP) is an autoimmune blistering disorder that occurs most frequently in patients older than 80 years [1,2]. The clinical characteristics include sub-epithelial blisters and pruritic urticarial plaques [3]. These symptoms occur due to autoantibodies against type XVII collagen in our basement membrane [1,3]. The autoimmune T-cell response consists of mostly immunoglobulin G (IgG) and immunoglobulin E (IgE) antibodies targeting our hemidesmosomal proteins [3,4]. These proteins are part of the dermo-epidermal junction and have been identified as BP180 and BP230 [3,4]. The neutrophil chemotaxis associated with our body’s immune response also contributes to the clinical symptoms seen with BP [4]. Drugs that alter the immune response or aspects of the epidermal basement membranes are triggers for this condition [5]. Other triggers include cytomegalovirus, Epstein-Barr virus, HHV-6, hepatitis B, hepatitis C, *Helicobacter pylori*, and *Toxoplasma gondii* [5]. Less common triggers include radiation therapy, ultraviolet radiation, thermal or electrical burns, surgical procedures, and transplants [6]. The current treatment for BP is systemic corticotherapy [7].

## Case presentation

The patient is a 38-year-old female with a past medical history of BP, lupus, epilepsy, asthma, granulomatosis with polyangiitis, and chronic obstructive pulmonary disease who presented to the emergency department via emergency medical services (EMS) with complaints of nausea and vomiting. She reported that nausea and vomiting started two days ago and have been gradually worsening since, with eight episodes of vomiting on the date of presentation. She was given ondansetron by EMS en route with minimal improvement. She also reported fever, diffuse abdominal pain, generalized weakness, and blisters on her left forearm. She denied urinary symptoms, diarrhea, chest pain, shortness of breath, cough, numbness, tingling, or other associated symptoms. She reported that her current symptoms were similar to the prior exacerbations of BP, which typically require admission for intravenous antibiotics due to her inability to tolerate oral intake. She reported being seen the day before for similar symptoms and being discharged on oral antibiotics. However, she had been unable to tolerate eating or drinking, or taking her antibiotics by mouth. She stated that she does not take steroids at home due to concern for avascular necrosis.

**Figure 1:**
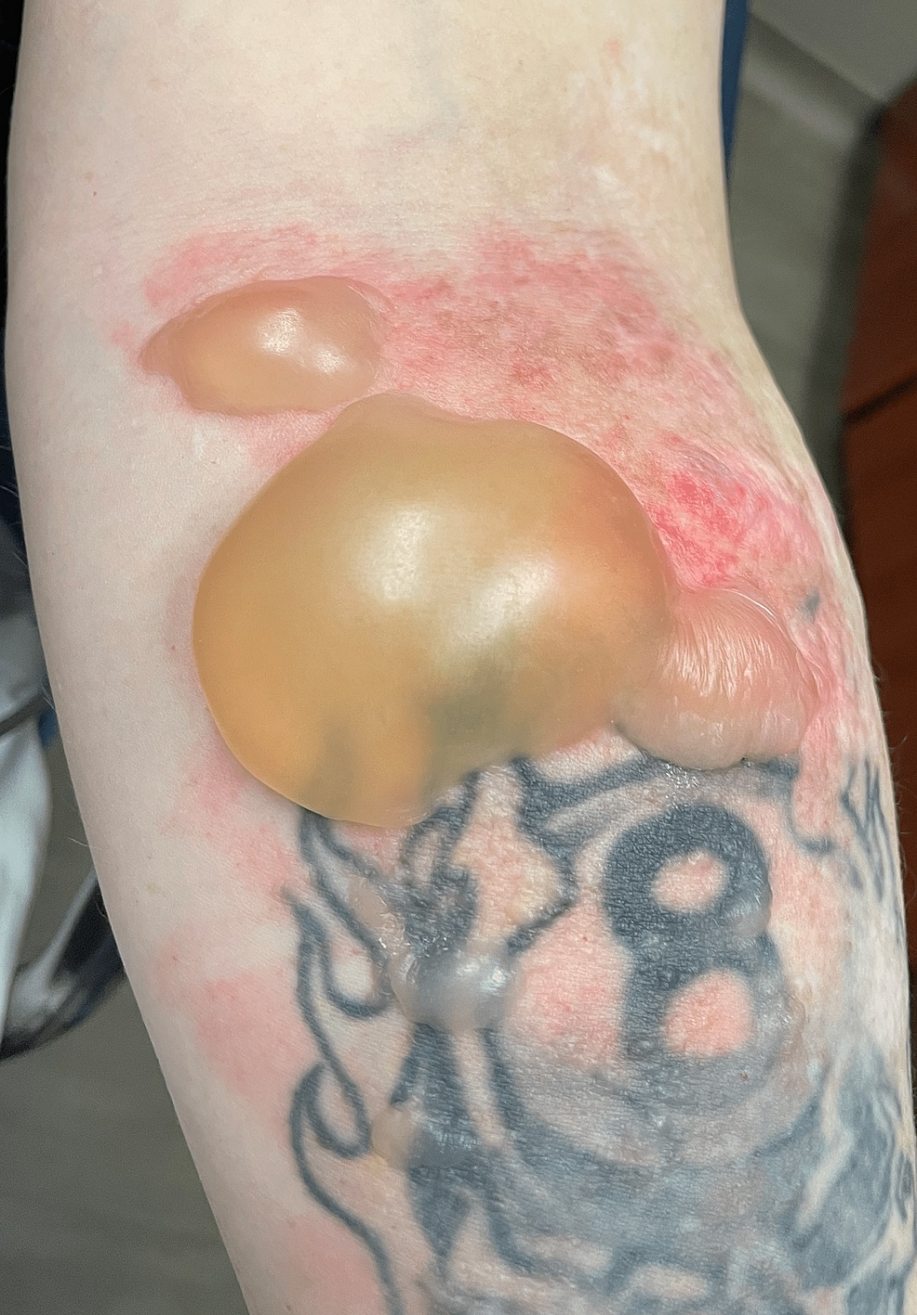
Photograph of the patient's arm demonstrating bullae

On examination, her vital signs were notable for tachycardia to 108 beats per minute but otherwise were within normal limits. Her examination was remarkable for a 5 cm × 4 cm bullous lesion on her left forearm with some surrounding erythema but no streaking. There were several smaller bullae surrounding the larger lesion (Figure 1).

The remainder of her physical examination was notable for some diffuse abdominal tenderness without rebound or guarding. Her workup was remarkable for an elevated white blood cell count of 17.2 × 10^3^cells/uL, elevated erythrocyte sedimentation rate of 46 mL/h, and an elevated C-reactive protein of 2.6 mg/L. Computed tomography of the abdomen and pelvis with contrast was remarkable for an incidental hypodensity seen within the left adnexal/ovarian region which may be reflective of a cyst. Pelvic ultrasound was negative for ovarian pathology. Due to concern for sepsis, blood cultures were obtained and the patient was started on vancomycin, clindamycin, and intravenous fluids and was admitted to the hospitalist service for further management of sepsis and failure of outpatient therapy. In the hospital, the patient's bullae drained spontaneously and she was started on methylprednisone and clobetasol for her BP flare. She was also given ondansetron and acetaminophen-hydrocodone for her nausea, vomiting, and pain. Through the duration of her hospitalization, the patient reported improvement of her symptoms and two days later was discharged to home on doxycycline, prednisone, and clobetasol. She was given outpatient follow-up with her primary care physician and rheumatology.

## Discussion

BP is an autoimmune blistering disorder that can have mortality as low as 11% at one year to as high as 40% at one year due to end-stage heart failure without proper treatment [8]. BP primarily affects the elderly and the current treatment regimen includes immunosuppressive medications [9]. While the use of corticosteroids is an effective treatment option for BP, often the medications themselves can poorly impact the health of the elderly leading to morbidity and mortality [9]. Hence, new treatment options are being explored for BP. The first one is dupilumab. Dupilumab is a medication that is often used for atopic dermatitis and presents as an antibody that targets the interleukin 4 receptor [9]. A recent case series treated 13 BP patients with dupilumab and found that symptoms improved in 92.3% of the patients (12 out of the 13) [10]. The medication helped clear the illness completely in 53.8% of the patients enrolled in the study (seven out of 13) [10]. There were no significant side effects reported as a result of this medication [10]. Furthermore, another medication that has been used as a treatment option is rituximab. In 2019, Polansky et al. found that 15 out of the 20 BP patients who had been put on rituximab therapy achieved remission within about six months (169 days) [11,12]. The patients who did not respond to rituximab either had persistent BP which required prednisone or responded to the second course of the medication and achieved remission about 10 months later instead of six months [11,12]. One patient from this study was lost to follow-up and none of the patients experienced life-altering side effects [11,12]. The major concern with this medication is that it causes depletion of B-cells which increases the risk of infection, especially in vulnerable patient populations such as the elderly [12]. It was deduced by the authors that more randomized control studies need to be done before rituximab can be confirmed as a safer alternative [12]. Finally, omalizumab is another drug that has been heavily investigated as a potential treatment option for BP patients. A study published in the *Journal of the American Academy of Dermatology* found that five out of the six patients who received omalizumab treatment for their BP experienced therapeutic benefits including a decrease in eosinophil count, lack of new bullae formation, less need for other immunosuppressants, and less pruritis [13]. This study followed the patients for 42 months and found that they had no major adverse effects from omalizumab treatment [13]. All in all, BP is a painful autoimmune blistering disorder that presents largely in the elderly who tend to respond negatively to the side effects of immunosuppressive treatments. Hence, it is important to continue to investigate new possible treatments for BP.

## Conclusions

This is a case of a 38-year-old woman who was adequately treated for her recurrent BP disease and superimposed infection. During her time as an inpatient, her bullae drained spontaneously, and she was treated for her sepsis and started on methylprednisolone and clobetasol for her BP flare. She responded well to the treatment regimen and was discharged within two days of admission. The patient may have additionally benefited from the off-label use of some of the current biologics. Research on dupilumab, rituximab, and omalizumab currently demonstrates symptom alleviation in patients with BP. Treatment of patients with a possible BP flare requires assessment for and treatment of possible superimposed infections in addition to careful use of anti-inflammatory or immunotherapy in patients who are highly susceptible to infection.
